# Extracellular matrix proteins produced by stromal cells in idiopathic pulmonary fibrosis and lung adenocarcinoma

**DOI:** 10.1371/journal.pone.0250109

**Published:** 2021-04-27

**Authors:** Mervi Kreus, Siri Lehtonen, Sini Skarp, Riitta Kaarteenaho

**Affiliations:** 1 Research Unit of Internal Medicine, University of Oulu, Oulu, Finland; 2 Medical Research Center Oulu, Oulu University Hospital, Oulu, Finland; 3 Department of Obstetrics and Gynecology, Oulu University Hospital, Oulu, Finland; 4 Northern Finland Birth Cohorts, Infrastructure for Population Studies, Faculty of Medicine, University of Oulu, Oulu, Finland; Medical University of South Carolina, UNITED STATES

## Abstract

Idiopathic pulmonary fibrosis (IPF) and lung cancer share common risk factors, epigenetic and genetic alterations, the activation of similar signaling pathways and poor survival. The aim of this study was to examine the gene expression profiles of stromal cells from patients with IPF and lung adenocarcinoma (ADC) as well as from normal lung. The gene expression levels of cultured stromal cells derived from non-smoking patients with ADC from the tumor (n = 4) and the corresponding normal lung (n = 4) as well as from patients with IPF (n = 4) were investigated with Affymetrix microarrays. The expression of collagen type IV alpha 1 chain, periostin as well as matrix metalloproteinase-1 and -3 in stromal cells and lung tissues were examined with quantitative real-time reverse transcriptase polymerase chain reaction and immunohistochemistry, respectively. Twenty genes were similarly up- or down-regulated in IPF and ADC compared to control, while most of the altered genes in IPF and ADC were differently expressed, including several extracellular matrix genes. Collagen type IV alpha 1 chain as well as matrix metalloproteinases-1 and -3 were differentially expressed in IPF compared to ADC. Periostin was up-regulated in both IPF and ADC in comparison to control. All studied factors were localized by immunohistochemistry in stromal cells within fibroblast foci in IPF and stroma of ADC. Despite the similarities found in gene expressions of IPF and ADC, several differences were also detected, suggesting that the molecular changes occurring in these two lung illnesses are somewhat different.

## Introduction

Idiopathic pulmonary fibrosis (IPF) is usually a lethal lung disease; patient survival after diagnosis varies around 3 years, thus being worse than that in many cancers, although similar to that in lung cancer [1, 2]. Indeed, IPF has been occasionally referred to as a malignant lung fibrosis. The principal underlying molecular mechanisms of IPF have remained unresolved and no effective curative pharmacological treatment is available [3].

IPF and lung cancer share common risk factors, like smoking, and patients with IPF have been shown to be at a greater risk to develop a lung carcinoma as compared to general population [4–6]. Furthermore, a tyrosine kinase inhibitor, namely nintedanib is used in the treatment of both IPF and lung cancer [7, 8] and moreover, myofibroblasts, are believed to act as principal pathogenetic cell types both in IPF and lung cancer [9, 10].

The gene expressions of IPF and lung cancer have been previously evaluated mostly with microarray studies performed on RNA isolated from whole lung tissues. A few studies have used cultured stromal cells derived from IPF and normal lung tissues (S1 Table) [11–16]. Stromal cells from lung cancer have been investigated in only one microarray analysis [17]. However, differences in gene expression profiles between cultured stromal cells derived from IPF and lung adenocarcinoma (ADC) have not been investigated previously in the same study.

Comparing IPF with lung cancer may provide clues to develop new therapeutic strategies for both diseases. Thus, we aimed to quantify the gene expressions of fibroblasts isolated from the patients with ADC from tumor and the corresponding normal lung as well as from IPF by microarray analysis. We wanted to evaluate even slight differences in gene expression of extracellular matrix (ECM) associated factors, also called as the matrisome, between (myo)fibroblasts derived from IPF, ADC and normal lung. The expression of collagen type IV alpha 1 chain (collagen α1(IV), gene name *COL4A1*), periostin (PN, gene name *POSTN*) as well as matrix metalloproteinase-1 (MMP-1, gene name *MMP1*) and matrix metalloproteinase-3 (MMP-3, gene name *MMP3*) were further studied with quantitative real-time reverse transcriptase polymerase chain reaction (qRT-PCR). Furthermore, the cell-specific expression of all of the above-mentioned ECM associated proteins was analyzed by immunohistochemistry (IHC).

## Materials and methods

### Patients, primary lung fibroblasts and lung tissue samples

Stromal cells were cultured from tumor and corresponding tumor-free peripheral lung of the ADC patients undergoing lung cancer-resection surgery (n = 4), and from surgical lung biopsy samples of the patients with IPF (n = 3) (Table 1) in Oulu University Hospital. In addition, one IPF sample was derived from peripheral lung outside the tumor from a patient operated for lung cancer (n = 1). Control samples were derived from histologically normal lung tissues from non-smoking patients being operated for lung cancer.

**Table 1:** Clinical characteristics of the study subjects examined in the microarray analysis.

Variables	IPF1	IPF2	IPF3	IPF4	ADC1	ADC2	ADC3	ADC4
Age (years)	63	71	72	68	70	79	74	78
Gender	Male	Male	Male	Male	Female	Male	Male	Female
Smoking status	Non-smoker	Ex-smoker	Smoker	Ex-smoker	Non-smoker	Non-smoker	Ex-smoker	Non-smoker
Pack-years	-	14	58	46	-	-	7*	-
FVC (% pred)	47	60	68	75	105	98	99	88
DL_CO_ (% pred)	43	62	62	54	73	98	87	80

ADC, lung adenocarcinoma; IPF, idiopathic pulmonary fibrosis; FVC, forced vital capacity; DL_CO_, single-breath carbon monoxide diffusing capacity; pred, predicted.

*between the ages 16 and 25.

Tissue samples were processed, and cells were cultured as described previously [18, 19]. Briefly, the cells were cultured in medium consisting of Minimum essential medium Eagle α modification (Sigma-Aldrich, Steinheim, Germany) supplemented with 13% heat-inactivated fetal bovine serum (FBS-Good, Pan Biotech, Aidenbach, Germany), 2 mM L-glutamine, 100 U/ml penicillin, 0.1 g/l streptomycin, 2.5 mg/l amphotericin B and 10 mM HEPES (all from Sigma-Aldrich). These cell lines are composed of both fibroblasts and myofibroblasts as previously described in our electron microscopic analyses [18, 19]. Cells were passaged at near confluency and used for experiments in passages 2–6. In the microarray and qRT-PCR analysis, cells were plated at density of 2000 cells /cm^2^ and cultured for 72 hours.

For IHC, lung tissue samples were obtained from the same patients whose cells were used in microarray-analysis. In addition, lung tissue samples were analyzed from surgical lung biopsies of 10 IPF patients as well as from 10 lung ADC patients undergoing cancer-resection surgery. Eight out of 9 normal peripheral lung samples were taken from the same patients who provided the cancer samples. Thus, 14 IPF, 14 ADC and 13 normal control samples were analyzed by IHC.

The Ethical Committee of Northern Ostrobothnia Hospital District in Oulu gave a favorable statement of the study protocol (64/2001, amendments 68/2005, 2/2008, 12/2014, 1/2015, 2/2018). All the patients gave their written informed consent. Paraffin embedded tissue samples have been approved for research use by National Supervisory Authority for Welfare and Health (reg. nr. 7323/05.01.00.06/2009 and 863/04/047/08).

### Microarray analysis

Total RNA was extracted using the RNeasy Mini Kit (Qiagen, Hilden, Germany) according to the manufacturer’s instructions and the concentration was measured using the NanoDrop spectrophotometry system (Thermo Fisher Scientific, Vilnius, Lithuania). The quality of RNA was analyzed with a Qiaxcel electrophoresis system (Qiagen). Microarray experiment was performed in Biocenter Oulu Sequencing Center core facility. Biotinylated cRNA were prepared from total RNA by using a 3’IVT Express Kit (Affymetrix Inc., Santa Clara, CA) according to the manufacturer’s instructions. After labeling, cRNA was hybridized to Affymetrix human hgu133Aplus2 chips. After hybridization, the microarray chip was washed and stained on an Affymetrix GeneChip Fluidics Station 450, according to the manufacturer’s instructions. The chips were then scanned using the Affymetrix GeneChip Scanner 3000 7G. An analysis of the microarray expression data was done using the R/Bioconductor through a graphical user interface, Chipster (3.12.5, CSC, Finland, http://chipster.csc.fi/) [20]. Normalization was performed using a custom chip description file for hgu133Aplus2. Intensity data were log_2_-transformed, and quantile normalized using robust multi-array average (RMA). Statistical analysis was performed with Chipster using empirical Bayes [21]. Benjamini-Hochberg was used as multiple correction for false discovery rate. Lists of differentially expressed genes between different groups were generated using a log_2_ fold change lower than -1 or higher than 1. Microarray data has been deposited in the Gene Expression Omnibus repository (http://www.ncbi.nlm.nih.gov/geo) with accession number GSE144338.

Differentially expressed genes were annotated using Matrisome annotator [22]. With this tool each entry is annotated as being or not being part of the matrisome and will be tagged with matrisome division (core matrisome vs matrisome-associated) and category (ECM glycoproteins, collagens, proteoglycans, ECM-affiliated proteins, ECM regulators, or secreted factors) [22].

### Quantitative real-time reverse transcriptase polymerase chain reaction

Quantitative RT-PCR was performed to confirm the expression of differentially expressed genes in the same primary lung fibroblast cells as used in the microarray analysis. Total RNA was extracted as described above in the Microarray analysis experiments. One-μg aliquots of RNA were reverse-transcribed using RevertAid First Strand cDNA Synthesis Kit (Thermo Fisher Scientific). PCR amplification was performed in triplicate as previously described [18], and the threshold cycle values were averaged. Reactions were performed by using iQTM SYBR Green Supermix (Bio-Rad Laboratories, Inc., USA). Non-template controls were included for each gene. Samples were processed for qRT-PCR using CFX Connect^TM^ Real-Time PCR Detection System (Bio-Rad Laboratories) in the following conditions: 95°C for 3 min, 40 cycles at 95°C for 10 s, annealing phase (temperature specific for each primer pair, see S2 Table) for 10 s and 72°C for 15 s, and a final extension phase of 72°C for 2 min. The melt curve was created in the following way: 81 cycles from 55°C with 0.5 degree increments every 5 s to 95°C. Relative gene expressions were quantified by using the 2^-ΔΔCT^ Livak method [23]. Gene expression levels were normalized to glyceraldehyde 3-phosphate dehydrogenase (*GAPDH*) or to hydroxymethylbilane synthase (*HMBS*) to confirm that the data are reproducible. The normalized values were compared to the average ΔC_T_ value of the control cell lines to calculate the fold changes. Two independent experiments were performed, and fold changes of each cell line were averaged.

### Immunohistochemistry

IHC stainings were performed in serial tissue sections. The antibodies used in this study are listed in S3 Table. Formalin-fixed and paraffin-embedded lung specimens were cut into 4-μm sections, de-paraffinized in xylene and rehydrated in a descending ethanol series. After microwave- or enzyme-stimulated antigen retrieval endogenous peroxidase was blocked with aqueous 0.3% H_2_O_2_ (Peroxidase-Blocking Solution, Dako, Glostrup, Denmark) for 10 min. Stainings were performed using Envision+ System Kit (Dako) with DAB 3,3’ diaminobenzidine chromogen. Counterstaining was performed with Mayer’s hematoxylin (Sigma-Aldrich). Phosphate-buffered saline, mouse and rabbit isotype controls (Invitrogen, Carlsbad, USA) were used as negative controls.

In order to identify the phenotype of the cells expressing collagen α1(IV), PN, MMP-1 and MMP-3, all cases were also studied for the markers of macrophages and monocyte lineage cells (cluster of differentiation 68, CD68), type II pneumocytes (thyroid transcription factor 1, TTF-1), endothelial cells (CD31) and myofibroblasts (alpha-smooth muscle actin, α-SMA) (S3 Table).

### Scoring of the immunoreactivity

The extent of the immunoreactivity for collagen α1(IV), PN, MMP-1 and MMP-3 was scored as negative (-) or positive (+) in stromal cells with widened alveolar tips, fibroblast foci and tumor stroma. Additionally, immunoreactivity was evaluated in alveolar epithelium, smooth muscle cells, alveolar macrophages, endothelial cells, epithelial cells of the bronchiole and cancer cells.

### Statistical analyses

Statistical analyses for qRT-PCR were performed by Statistical Package for the Social Sciences (SPSS; version 25.0, Chigago, IL, USA) using Mann Whitney U test when comparing IPF and control samples and Wilcoxon Signed Rank Test when comparing ADC and control samples. Values of P<0.05 were considered as significant.

## Results

### The different mRNA expression of several ECM genes in stromal cells of IPF, ADC and normal lung

**Fig 1:**
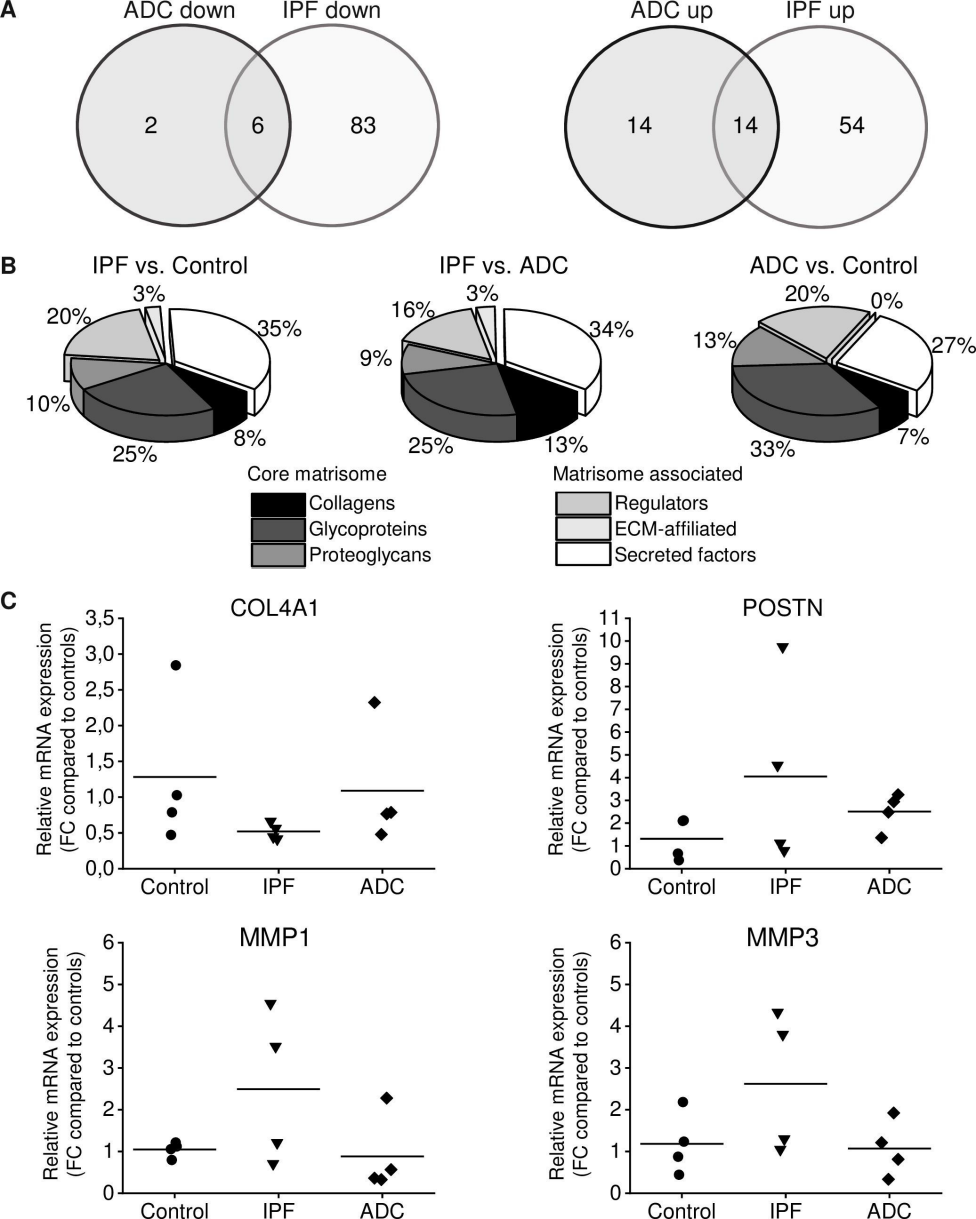
Gene expression differences between IPF, ADC and normal control lung. Total RNA was isolated from stromal cells cultured from lung adenocarcinoma (ADC, n = 4) and the corresponding histologically normal control lung (n = 4) as well as from idiopathic pulmonary fibrosis (IPF) patients (n = 4); the specimens were subjected to microarray analysis and quantitative real-time reverse transcriptase polymerase chain reaction analysis (qRT-PCR). (A) Venn diagram showing the number of either up- or down-regulated genes in stromal cells derived from IPF and ADC compared to control cells. (B) Number of differentially expressed genes in different matrisome categories between IPF, ADC and normal control lung. (C) Validation of genes of interest by qRT-PCR analysis. Data were normalized to glyceraldehyde 3-phosphate dehydrogenase (*GAPDH*) and represented graphically as the fold change (FC) as compared to control cell lines. Data are presented as means with each dot representing an individual cell line. The mRNA expression of *COL4A1* was lower while the mRNA expression of *MMP1* and *MMP3* was higher in cells derived from IPF compared to cells derived from ADC or control. *POSTN* expression was higher in both IPF and ADC derived cells compared to control.

Using an Affymetrix platform (U133Aplus2), we determined gene expression levels in fibroblasts prepared from lung tissue of 4 patients with IPF, 4 patients with ADC as well as in 4 control samples consisting of histologically normal looking lung collected outside of the tumor. We observed modest differences between groups, but due to the small sample size, statistical significances were not achieved. With our selection criteria (log_2_ fold change lower than -1 or higher than 1) 36 genes between primary fibroblasts derived from ADC and normal lung, 157 genes between IPF and normal lung and 152 genes between IPF and ADC were differentially expressed (S4–S6 Tables). Fourteen genes (*LINC01116*, *GPNMB*, *CSTA*, *S100A4*, *CHI3L1*, *TIMP3*, *VAMP8*, *HOXC6*, *MEOX2*, *CD36*, *POSTN*, *CRLF1*, *SERPINF1*, *ST6GALNAC5*) were up-regulated in both IPF and ADC and six genes (*CHRDL1*, *TRIM55*, *ACAN*, *CHRM2*, *PCOLCE2*, *RARRES1*) were down-regulated in both IPF and ADC in comparison to control (Fig 1A). Forty matrisome genes were altered in IPF and 15 in ADC as compared to control. Matrisome genes annotated with Matrisome Annotator [22] are shown in Fig 1B and Tables 2–4. Of the matrisome genes *COL4A1* was down-regulated in IPF compared to ADC and control, *POSTN* was up-regulated in IPF and ADC compared to control, *MMP1* and *MMP3* were up-regulated in IPF compared to ADC and control, and because of their differential expression profiles they were chosen for the present research.

**Table 2:** Differentially expressed matrisome genes in stromal cells derived from IPF compared to normal lung.

Matrisome division	Matrisome category	Gene symbol	Gene name	Log_2_FC IPF/Control
Core matrisome	Collagens	*COL10A1*	collagen, type X, alpha 1	1.20
		*COL15A1*	collagen, type XV, alpha 1	-1.11
		*COL4A1*	collagen, type IV, alpha 1	-1.03
	ECM Glycoproteins	*CRISPLD2*	cysteine-rich secretory protein LCCL domain containing 2	-1.37
		*EFEMP1*	EGF containing fibulin-like extracellular matrix protein 1	-1.24
		*FBN2*	fibrillin 2	1.62
		*FGL2*	fibrinogen-like 2	-1.01
		*MATN2*	matrilin 2	-1.05
		*MGP*	matrix Gla protein	-1.64
		*NTN4*	netrin 4	-1.64
		*PCOLCE2*	procollagen C-endopeptidase enhancer 2	-1.24
		*POSTN*	periostin, osteoblast specific factor	1.03
		*TNFAIP6*	tumor necrosis factor, alpha-induced protein 6	1.15
	Proteoglycans	*ACAN*	aggrecan	-1.10
		*BGN*	biglycan	-1.05
		*DCN*	decorin	-1.42
		*LUM*	lumican	-1.18
Matrisome-associated	ECM Regulators	*ADAMTS5*	ADAM metallopeptidase with thrombospondin type 1 motif, 5	-1.03
		*CSTA*	cystatin A (stefin A)	1.56
		*MMP1*	matrix metallopeptidase 1	1.50
		*MMP10*	matrix metallopeptidase 10	2.00
		*MMP3*	matrix metallopeptidase 3	3.10
		*PRSS3*	protease, serine, 3	1.00
		*SERPINF1*	serpin peptidase inhibitor, clade F (alpha-2 antiplasmin, pigment epithelium derived factor), member 1	1.89
		*TIMP3*	TIMP metallopeptidase inhibitor 3	1.02
	ECM-affiliated Proteins	*CLEC14A*	C-type lectin domain family 14, member A	1.09
	Secreted Factors	*BRINP3*	bone morphogenetic protein/retinoic acid inducible neural-specific 3	-1.68
		*CHRDL1*	chordin-like 1	-2.22
		*CRLF1*	cytokine receptor-like factor 1	1.73
		*CXCL12*	chemokine (C-X-C motif) ligand 12	-1.15
		*CXCL3*	chemokine (C-X-C motif) ligand 3	1.10
		*EREG*	epiregulin	1.53
		*HHIP*	hedgehog interacting protein	-1.18
		*IFNE*	interferon, epsilon	1.21
		*IL12A*	interleukin 12A	1.05
		*IL24*	interleukin 24	2.61
		*IL6*	interleukin 6	-1.04
		*NTF3*	neurotrophin 3	-1.08
		*PTN*	pleiotrophin	-1.11
		*S100A4*	S100 calcium binding protein A4	2.56

List of differentially expressed genes between IPF and control was generated using log_2_FC lower than -1 or higher than 1 and the differentially expressed genes were annotated with Matrisome annotator [22]. ECM, extracellular matrix; IPF, idiopathic pulmonary fibrosis; log_2_FC, log_2_ fold change.

**Table 3:** Altered matrisome-associated genes in stromal cells derived from lung ADC compared to normal lung.

Matrisome division	Matrisome category	Gene symbol	Gene name	Log_2_FC ADC/Control
Core matrisome	Collagens	*COL11A1*	collagen, type XI, alpha 1	1.33
	ECM Glycoproteins	*MFAP5*	microfibrillar associated protein 5	1.31
		*PCOLCE2*	procollagen C-endopeptidase enhancer 2	-1.13
		*POSTN*	periostin, osteoblast specific factor	1.70
		*RELN*	reelin	-1.63
		*RSPO3*	R-spondin 3	1.02
	Proteoglycans	*ACAN*	aggrecan	-1.06
		*HAPLN1*	hyaluronan and proteoglycan link protein 1	1.22
Matrisome-associated	ECM Regulators	*CSTA*	cystatin A (stefin A)	1.97
		*SERPINF1*	serpin peptidase inhibitor, clade F (alpha-2 antiplasmin, pigment epithelium derived factor), member 1	1.16
		*TIMP3*	TIMP metallopeptidase inhibitor 3	1.29
	Secreted Factors	*CHRDL1*	chordin-like 1	-1.21
		*CRLF1*	cytokine receptor-like factor 1	1.47
		*S100A4*	S100 calcium binding protein A4	1.22
		*TNFSF4*	tumor necrosis factor (ligand) superfamily, member 4	1.02

List of differentially expressed genes between ADC and control was generated using log_2_FC lower than -1 or higher than 1 and the differentially expressed genes were annotated with Matrisome annotator [22]. ADC, adenocarcinoma; ECM; extracellular matrix; log_2_FC, log_2_ fold change.

**Table 4:** Differentially expressed matrisome-associated genes in stromal cells derived from IPF compared to lung ADC.

Matrisome division	Matrisome category	Gene symbol	Gene name	Log_2_FC IPF/ADC
Core matrisome	Collagens	*COL11A1*	collagen, type XI, alpha 1	-1.97
		*COL15A1*	collagen, type XV, alpha 1	-1.34
		*COL4A1*	collagen, type IV, alpha 1	-1.18
		*COL4A2*	collagen, type IV, alpha 2	-1.02
	ECM Glycoproteins	*EDIL3*	EGF-like repeats and discoidin I-like domains 3	-1.25
		*EFEMP1*	EGF containing fibulin-like extracellular matrix protein 1	-1.02
		*FBN2*	fibrillin 2	1.05
		*IGFBP7*	insulin-like growth factor binding protein 7	-1.19
		*MFAP5*	microfibrillar associated protein 5	-1.30
		*MGP*	matrix Gla protein	-1.64
		*NTN4*	netrin 4	-1.74
		*THBS2*	thrombospondin 2	-1.07
	Proteoglycans	*DCN*	decorin	-1.08
		*HAPLN1*	hyaluronan and proteoglycan link protein 1	-1.01
		*LUM*	lumican	-1.31
Matrisome-associated	ECM Regulators	*MMP1*	matrix metallopeptidase 1	2.44
		*MMP10*	matrix metallopeptidase 10	1.61
		*MMP3*	matrix metallopeptidase 3	2.40
		*PRSS3*	protease, serine, 3	1.37
		*SULF1*	sulfatase 1	-1.49
	ECM-affiliated Proteins	*CLEC14A*	C-type lectin domain family 14, member A	1.04
	Secreted Factors	*BRINP3*	bone morphogenetic protein/retinoic acid inducible neural-specific 3	-1.30
		*CHRDL1*	chordin-like 1	-1.01
		*CXCL3*	chemokine (C-X-C motif) ligand 3	1.20
		*CXCL5*	chemokine (C-X-C motif) ligand 5	1.00
		*EREG*	epiregulin	1.10
		*IFNE*	interferon, epsilon	1.20
		*IL24*	interleukin 24	1.80
		*IL6*	interleukin 6	-1.23
		*MEGF6*	multiple EGF-like-domains 6	-1.08
		*NTF3*	neurotrophin 3	-1.17
		*S100A4*	S100 calcium binding protein A4	1.35

List of differentially expressed genes between IPF and ADC was generated using log_2_FC lower than -1 or higher than 1 and the differentially expressed genes were annotated with Matrisome annotator [22]. ADC, adenocarcinoma; ECM, extracellular matrix; IPF, idiopathic pulmonary fibrosis; log_2_FC, log_2_ fold change.

Microarray results were confirmed by qRT-PCR on selected ECM genes *COL4A1*, *POSTN*, *MMP1* and *MMP3*. These factors were differentially expressed between IPF, ADC and normal lung and they might have a role in fibrosis [24–27]. The mRNA levels of *COL4A1*, *POSTN*, *MMP1* and *MMP3* normalized to those of *GAPDH* (Fig 1C) or *HMBS* (S1 Fig) showed a trend in the direction of a change observed in the microarray results although statistical significance was not achieved due to the small sample size. As compared to controls, *COL4A1* mRNA expressions were 41% in IPF and 85% in ADC when those of *POSTN* in IPF and ADC were 308% and 191%, respectively. *MMP1* and *MMP3* expressions in IPF were 238% and 221%, respectively, and in ADC 84% and 90%, respectively, compared to control.

### Collagen α1(IV), PN, MMP-1 and MMP-3 were localized in stromal cells of IPF and ADC in immunohistochemistry

IHC was used to confirm the expression of collagen α1(IV), PN, MMP-1 and MMP-3 at the protein level in stromal cells within lung tissues. In particular, we evaluated the expression of these ECM proteins in fibroblast foci of IPF, tumor stroma of ADC, and widened alveolar tips of normal lung, since these areas contain abundant fibroblasts and myofibroblast (Table 5). Widened alveolar tips were defined as widened endings of free interalveolar septa as previously described [28]. S2 Fig shows the IHC expression of markers of macrophages and monocyte lineage cells (CD68), type II pneumocytes (TTF-1), endothelial cells (CD31) and myofibroblasts (α-SMA).

**Table 5:** Scoring of the immunohistochemical intensity of collagen α1(IV), PN, MMP-1 and MMP-3 in IPF, ADC and normal control lung*.

Diagnosis/Number of Cases	Normal alveolar epithelium	Widened alveolar tips	Fibroblast foci	Hyperplastic alveolar epithelium	Tumor stroma	Tumor cells	Bronchial Epithelium	Smooth muscle cells	Endothelium	Macrophages
**Collagen α1(IV)**										
Control/13	+b/13	+/13	n/13	n/13	n/13	n/13	-/9, +/4	+/13	+b/13	-/9, +/4
IPF/14	+b/10, n/4	+/10, n/4	+/14	-/8, +/6	n/14	n/14	-/10, +/4	+/14	+b/14	-/9, +/5
ADC/14	+b/11, n/3	+/11, /3	n/14	n/14	n/14	-/10, +/14	-/9, +/2, n/3	+/10, n/4	+b/14	-/11, +/3
**PN**										
Control/13	+/13	+/13	n/13	n/13	n/13	n/13	-/13	-/13	-/4, +/9	-/12, +/1
IPF/14	+/10, /4	+/10, n/4	+/14	-/14	n/14	n/14	-/14	-/13, +/1	-/4, +/10	-/14
ADC/14	+/11, n/3	+/11, n/3	^b^/14	n/14	+/14	-/14	-/10, n/4	-/10, n/4	-/5, +/9	-/13, +/1
**MMP-1**										
Control/13	-/1, +/12	-/3, +/10	n/13	n/13	n/13	n/13	+/13	-/1, +/12	+/13	+/13
IPF/14	+/10, n/4	-/1, +/9, n/4	+/14	-/1, +/13	n/14	n/14	+/13, n/1	-/4, +/6, n/4	-/1, +/13	+/14
ADC/14	+/11, n/3	+/11, n/3	n/14	n/14	+/14	+/14	+/10, n/4	-/10, +/4	+/14	+/14
**MMP-3**										
Control/13	+/13	-/3, +/10	n/13	n/13	n/13	n/13	-/1, +/12	-/9, +/4	-/1, +/12	+/13
IPF/14	+/10, n/4	-/2, +/8, n/4	-/3, +/11	+/14	n/14	n/14	+/14	-/8, +/6	+/14	+/14
ADC/14	+/11, n/3	-/1, +/10, n/3	n/14	n/14	+/14	+/14	+/10, n/4	-/7, +/3, n/4	+/14	+/14

ADC, adenocarcinoma; b, immunoreactivity in basement membrane; IPF, idiopathic pulmonary fibrosis; MMP, matrix metalloproteinase; n, structure not present; PN, periostin; -, negative immunoreactivity; +, positive immunoreactivity.

* Score/number of cases.

**Fig 2:**
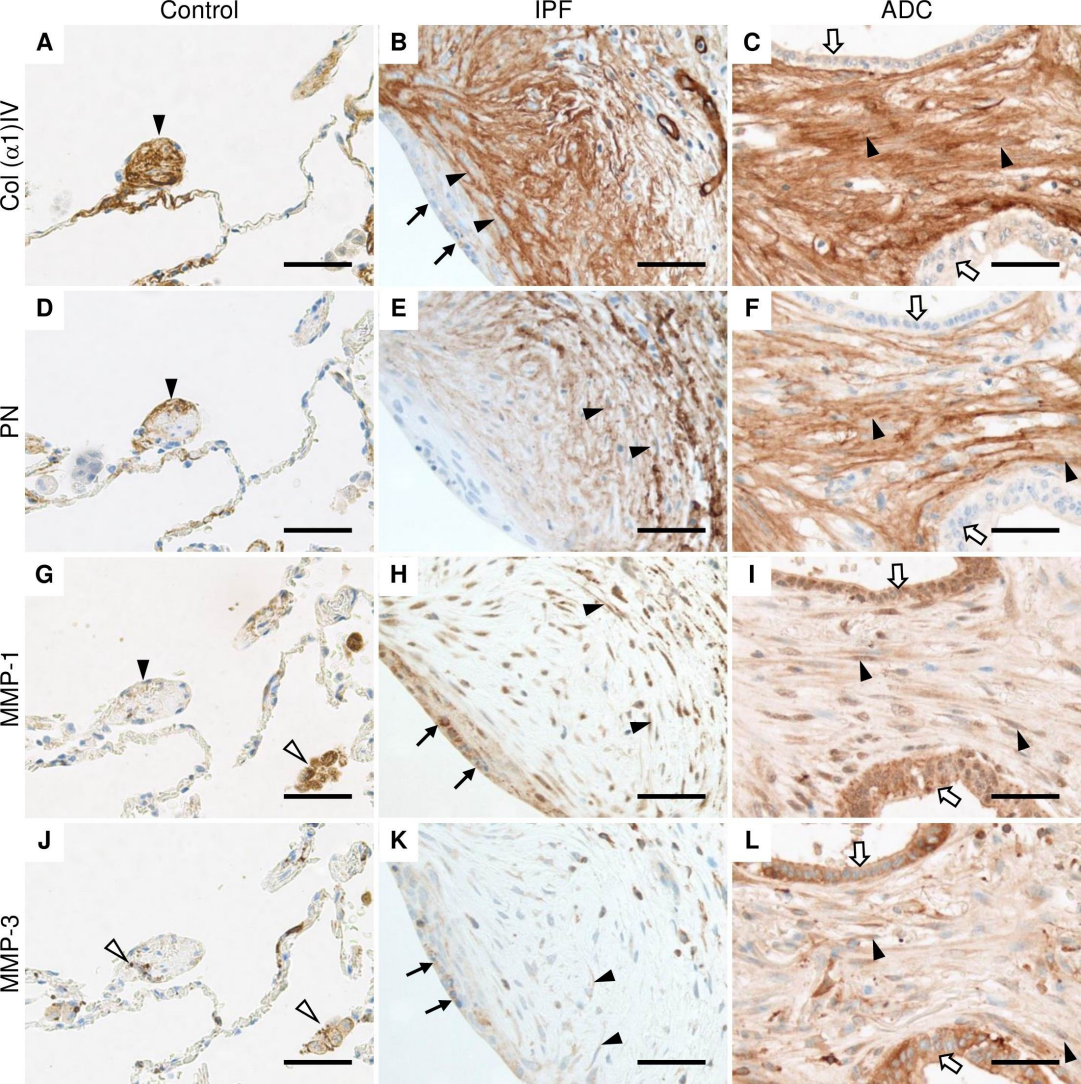
Immunohistochemical localization of Col α1(IV), PN, MMP-1 and MMP-3 in normal control lung, IPF and ADC. Sections were obtained from patients with lung adenocarcinoma (ADC) from both the tumor area and histologically normal lung as well as from patients with idiopathic pulmonary fibrosis (IPF). (A, B and C) Stromal cells of control lung, IPF and ADC were strongly positive for collagen (Col) α1(IV) chain (black arrow heads), while hyperplastic alveolar epithelial cells lining fibroblast focus (black arrows) and cancer cells (white arrows) were weakly positive. (D, E, and F) Stromal cells of control lung, IPF and ADC were positive for periostin (PN) (black arrow heads). (G) Alveolar macrophages (white arrow heads) were strongly positive for matrix metalloproteinase (MMP) -1, while cells with a widened alveolar tip of normal lung were weakly positive (black arrowhead). (H) Stromal cells (black arrow heads) and epithelial cells lining fibroblast foci (black arrows) in IPF were positive for MMP-1. (I) Cancer cells (white arrows) and stromal cells (black arrow heads) of ADC were positive for MMP-1. (J) Alveolar macrophages and monocyte lineage cells (white arrow heads) were positive for MMP-3 in normal lung. (K) Hyperplastic alveolar epithelial cells (black arrows) and some stromal cells (black arrow heads) were positive for MMP-3 in IPF. (L) Stromal cells (black arrow heads) as well as cancer cells (white arrows) were positive for MMP-3. Scale bar 50 μm.

Strong expression of collagen α1(IV) was observed extracellularly within stromal cells of the widened alveolar tips in control lung (Fig 2A). Collagen α1(IV) was also localized in the basement membranes of alveolar epithelium and endothelium, smooth muscle cells and in a few cases, a weak expression was observed in bronchiolar epithelial cells and alveolar macrophages. In IPF, collagen α1(IV) was expressed extracellularly in the stromal cells of fibroblast foci, while a very weak immunoreactivity was observed in the hyperplastic alveolar epithelium lining fibroblast foci (Fig 2B). A strong collagen α1(IV) expression was observed within stromal cells in ADC while a weak expression was detected in the cancer cells in a few cases (Fig 2C).

PN was observed extracellularly in some widened alveolar tips of normal control lungs (Fig 2D), within alveolar epithelial cells and the bronchiolar basement membrane zone, but not in bronchiolar epithelial cells, smooth muscle cells or alveolar macrophages. In IPF, the strongest expression of PN was observed in stromal cells within fibroblast foci (Fig 2E). In ADC, PN was observed in tumor stroma, but not in the cancer cells (Fig 2F).

A very weak extracellular MMP-1 immunoreactivity was observed occasionally in widened alveolar tips and smooth muscle cells in controls (Fig 2G) when a strong MMP-1 expression was detected in the alveolar macrophages, some alveolar epithelial cells, endothelial cells, and bronchiolar epithelium. Some stromal cells within fibroblast foci were positive for MMP-1 in IPF (Fig 2H) with the strongest MMP-1 expression being observed in the hyperplastic alveolar epithelial cells lining fibroblast foci. Both cancer cells and stromal cells were positive for MMP-1 in ADC (Fig 2I).

Almost negative expression of MMP-3 was detected in some of the widened alveolar tips within normal control lung (Fig 2J). MMP-3 was mainly expressed in alveolar macrophages and monocyte lineage cells, and bronchiolar epithelial cells. Weak MMP-3 expression was observed in some alveolar epithelial cells and smooth muscle cells. Some stromal cells of fibroblast foci were positive for MMP-3 in IPF (Fig 2K) and in addition, hyperplastic alveolar epithelial cells were positive. Both cancer cells and stromal cells were mainly positive for MMP-3 in ADC (Fig 2L).

## Discussion

We investigated mRNA expressions in stromal cell lines derived from IPF, ADC and normal lung by microarray analysis and we observed that 20 genes were similarly up- or down-regulated in IPF and ADC as compared to control, whereas most of the altered genes in IPF and ADC were different, including several ECM genes. We were particularly interested in the ECM associated genes and selected *COL4A1*, *POSTN*, *MMP1* and *MMP3* since they have been previously shown to be associated with pulmonary fibrosis. Furthermore, most of the previous studies have focused on epithelial cells, and not on stromal cells as investigated here. We concentrated on those genes that revealed a differential expression between IPF and lung cancer, such as higher expression of *MMP1* and *MMP3* in IPF than ADC, and lower expression of *COL4A1* in IPF than in ADC. Because the expression of these factors in lung cancer and IPF has not been previously compared in the same study, we believe that this kind of protocol may provide novel information. *POSTN* was chosen as an example of a gene being equally highly expressed both in IPF and lung ADC.

Previous studies have revealed differences in gene expression between IPF and lung cancer [29–31]. A study comparing gene expression in lung tissues by microarray analysis from five patients with both lung cancer and IPF revealed five genes e.g. *SMAD4*, *P21*, *MT1A*, *MMP7* and *TIMP1*; these were down-regulated in cancer tissue in comparison to IPF, in at least two of the patients [29]. Cancer associated lung fibroblasts have been previously compared to paired normal fibroblasts; in that study, 46 differentially expressed genes were identified [17]. Some of these genes e.g. *CHI3L1*, *ST6GALNAC5*, *COL11A1*, *MFAP5*, *TNFSF4* were identified also in our analysis as differentially expressed genes between ADC and control or between IPF and control suggesting that at least some disease related changes in the transcriptome are maintained during *in vitro* culture.

A previous study identified 178 differentially expressed genes in stromal cells derived from IPF and normal lung [14]. Fourteen of these genes were also differentially expressed in our comparative analysis between IPF and control, and two of these genes (*S100A4* and *POSTN*) were also differentially expressed between ADC and controls. Another study identified 547 genes which were differentially expressed in IPF compared to controls [13]. Thirty-nine of these genes were also differentially expressed in our microarray analysis, but not all in the same direction since only 14 genes were similarly up- or down-regulated in IPF when compared to our study. Rodriguez *et al*. did not identify any statistically significant gene expression differences between cultured fibroblasts derived from either IPF or normal lung, but they observed that the gene expression of cultured fibroblasts differed from that of non-cultured, freshly isolated fibroblasts [16].

Our study identified a down-regulation of *COL4A1* in IPF derived stromal cells in comparison to control cells and ADC. This finding is different from published microarray-based and RNA sequencing studies, in which *COL4A1* was found as an up-regulated gene in fibroblasts and tissues derived from IPF patients as compared to control [13, 32]. Collagen IV expression has been shown to localize in the fibroblast foci and early fibrotic lesions of IPF [24, 33], findings which are supported by our IHC analysis which detected intense collagen α1(IV) immunoreactivity in the surrounding spindle shaped cells within fibroblast foci. *COL4A1* expression in lung cancer has been previously detected in stromal fibroblasts surrounding tumor cells by mRNA *in situ* hybridization [34] and discontinuous collagen α1(IV) protein expression has been detected around well-differentiated clusters in ADCs [35], results which are similar to the present findings i.e. strong collagen α1(IV) immunoreactivity in the stroma of ADC. Based on the results of our study one can speculate that the (myo)fibroblasts might have different abilities to produce collagen α1(IV) in IPF and ADC.

PN has been previously studied in both IPF and lung cancer, and *POSTN* up-regulation in both cultured and non-cultured stromal cells derived from IPF as compared to normal lung fibroblasts has been reported [15, 36]. The gene expression level of *POSTN* has been shown to be higher also in IPF lung tissues as compared to normal lungs [37, 38]. We confirmed the previous findings showing that PN mRNA and protein were up-regulated in both IPF and ADC derived stromal cells. Similarly to our results, PN immunoreactivity has been observed in areas of active fibrosis and fibroblast foci in IPF [25, 39]. *POSTN* gene expression has been claimed to be up-regulated in lung cancer tissues compared to normal lung tissue [40], and it has been detected by IHC, in lung cancer stroma but not in cancer cells, in support of our findings [41, 42]. However, it is still a matter of debate whether the source of PN is cancer cells or stromal cells surrounding cancer tissues. Some investigators have claimed that the mRNA expression of *POSTN* occurs only in stromal cells [43], or in cancer cells [44]. In lung cancer, *POSTN* mRNA expression has been detected mainly in the stromal cells surrounding the cancer cells, whereas very little expression was found in the cancer cells themselves [45].

We observed that *MMP1* was up-regulated in stromal cells from IPF compared to ADC, which is a novel finding. *MMP1* gene expression has also been previously shown to be up-regulated in non-cultured fibroblasts derived from IPF lung [36]. Several microarray-based studies have revealed that *MMP1* gene expression was up-regulated in IPF in comparison to normal lung tissues [37, 46–48]. *MMP1* gene expression has been shown to be up-regulated also in ADC and squamous cells carcinoma tissues as compared to normal lung [49]. Previous IHC studies have shown that MMP-1 was expressed mainly in epithelial cells and alveolar macrophages in IPF [48, 50]. In turn, we observed MMP-1 immunoreactivity also in stromal cells in IPF and ADC, which results were in line with previous reports [51, 52].

Several microarray-based studies have reported up-regulated *MMP3* gene expression lung tissues derived from IPF patients [38, 46]. In normal lung, MMP-3 was mainly expressed by alveolar macrophages while in IPF, it was expressed in macrophages, epithelial cells, intravascular leukocytes and fibroblasts [27, 38]. Our microarray analysis did not identify any differences in *MMP3* gene expression between stromal cells derived from ADC and normal lung, at odds with a previous study in which an increased *MMP3* gene expression in ADC and squamous cells carcinoma was observed [45]. We observed also MMP-3 immunoreactivity in both tumor cells and stromal cells whereas previous studies have shown stronger MMP-3 expression in tumor cells than in stromal cells in ADC [52, 53].

The phenotypic changes in the fibroblasts during their passage in culture and differences in the culture methods might have affected the results as was previously speculated [16]. Certain disease specific alterations in gene expressions are not maintained during cell culture conditions and the disappearance of these differences could be enhanced if cell culture conditions such as cell confluency are not controlled. Therefore, we adopted stringently controlled conditions from the very start of the sample collection. The most serious limitation of the study was the small number of cell lines examined in the microarray study. It is notable, however, that the numbers of cell lines in the previous published studies have also been limited [11–16]. A recent single-cell RNA-sequencing study showed that human lung fibroblasts are a very heterogeneous population [54]. The heterogeneity of the original cell populations before culture may also explain the differences between microarray studies.

Despite the similarities found in gene expressions of IPF and lung ADC, there were also several differences, suggesting that the molecular changes occurring in these two lung illnesses are at least partly different. The comparison of IPF and lung cancer may, however, reveal new information about the pathogenesis of these severe diseases, which may help to develop new therapies for patients.

## Supporting information

S1 Fig Validation of genes of interest by quantitative real-time reverse transcriptase polymerase chain reaction analysis similar to Fig 1C, but with data for each target gene normalized to *HMBS (*hydroxymethylbilane synthase). Data are represented graphically as the fold changes (FC) as compared to control cell lines. Lines represent means and each dot represents an individual cell line. ADC, adenocarcinoma; IPF, idiopathic pulmonary fibrosis.
(PDF)

S2 Fig Immunohistochemical localization of α-SMA, TTF-1, CD68 and CD31 in normal control lung, IPF and ADC. (A, B, and C) Spindle shaped cells (black arrowheads) within widened alveolar tips of normal control lung, fibroblast foci of idiopathic pulmonary fibrosis (IPF) lung and stroma of lung adenocarcinoma (ADC) are positive for alpha smooth muscle actin (α-SMA). (D) Some alveolar epithelial cells (black arrows) in normal lung are positive for thyroid transcription factor 1 (TTF-1), which is a marker for type two pneumocytes. (E) Epithelial cells lining fibroblast focus of IPF lung are positive for TTF-1. (F) In lung ADC, cancer cells and cancer stroma are negative for TTF-1. (G) Figure shows cluster of differentiation (CD) 68 positive alveolar macrophages and monocyte lineage cells in normal lung. (H and I) Some cells within the fibroblast focus and cancer stroma are positive for CD68 (white arrowhead), which stains macrophages and monocyte lineage cells. (J, K and L) Figure shows CD31 positive endothelial cells (white arrows) lining capillaries within normal lung, fibroblast foci of IPF lung and stroma of ADC. (M, N and O) Negative controls where primary antibody was substituted with rabbit isotype control, mouse isotype control or phosphate buffered saline (PBS), respectively. Scale bar 50 μm.
(PDF)

S1 Table Microarray studies using cultured stromal cells derived from IPF or ADC. (DOCX)

S2 Table Sequences, annealing temperatures and amplicon sizes of quantitative RT-PCR primers used in this study. (DOCX)

S3 Table Antibodies used for immunohistochemistry in this study. (DOCX)

S4 Table Differentially expressed genes in IPF compared to control. All up- or down-regulated genes (log_2_FC higher than 1 or lower than -1) in stromal cells derived from patients with IPF compared to normal control lung.
(DOCX)

S5 Table Differentially expressed genes in IPF compared to ADC. All up- or down-regulated genes (log_2_FC higher than 1 or lower than -1) in stromal cells derived from patients with IPF compared to ADC.
(DOCX)

S6 Table Differentially expressed genes in ADC compared to control. All up- or down-regulated genes (log_2_FC higher than 1 or lower than -1) in stromal cells derived from ADC compared to normal control lung.
(DOCX)
