# Supplementary material for: Extracellular matrix proteins produced by stromal cells in idiopathic pulmonary fibrosis and lung adenocarcinoma
Source: PLoS One. 2021 Apr 27;16(4):e0250109. doi: 10.1371/journal.pone.0250109 (PMC8078755; doi:10.1371/journal.pone.0250109)
Supplement: S1 Fig — Data are represented graphically as the fold changes (FC) as compared to control cell lines. Lines represent means and each dot represents an individual cell line. ADC, adenocarcinoma; IPF, idiopathic pulmonary fibrosis. (PDF) [file pone.0250109.s001.pdf]

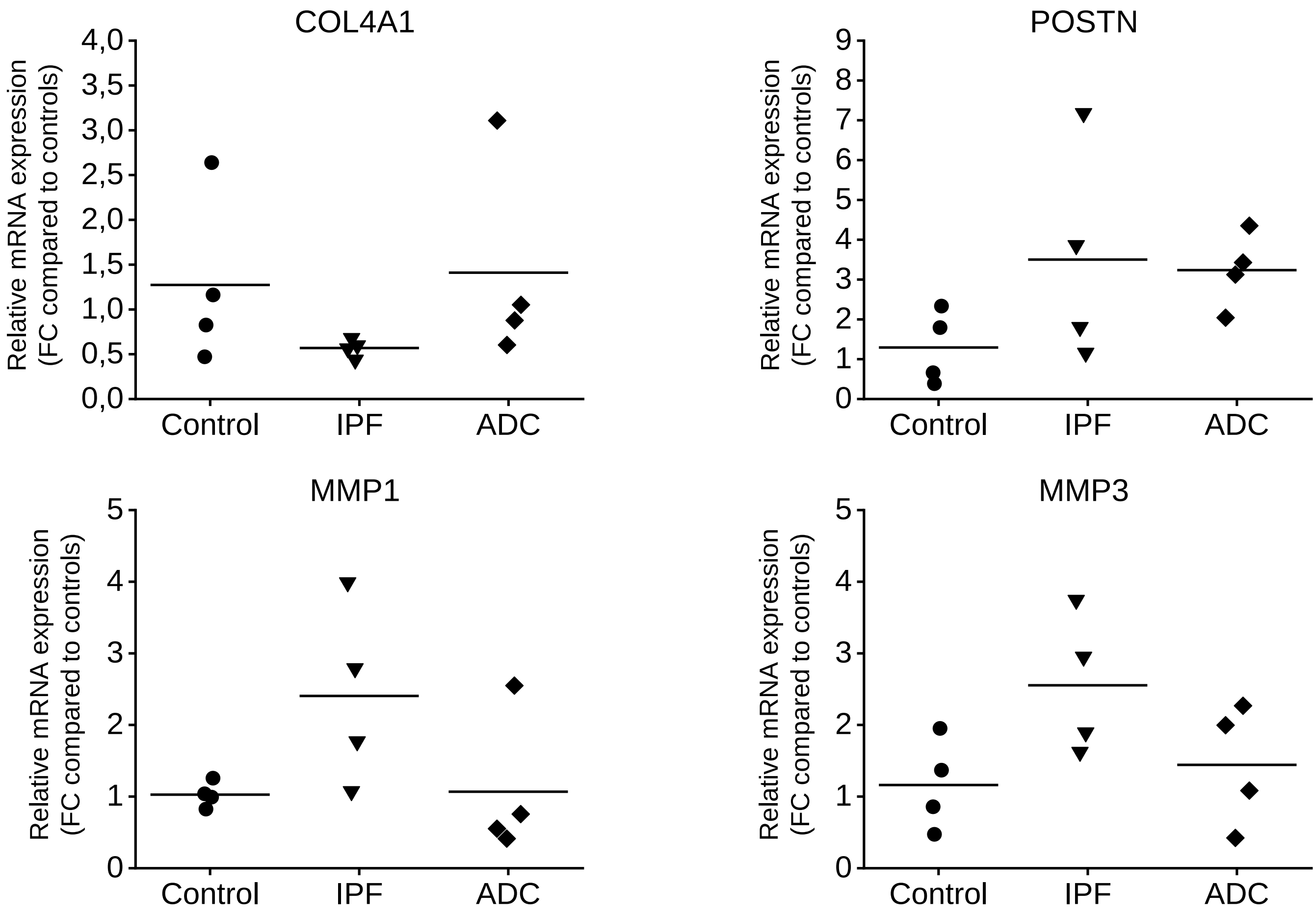

S1 Fig. Validation of genes of interest by quantitative real-time reverse transcriptase polymerase chain reaction analysis similar to Fig 1C, but with data for each target gene normalized to *HMBS* (hydroxymethylbilane synthase). Data are represented graphically as the fold changes (FC) as compared to control cell lines. Lines represent means and each dot represents an individual cell line. ADC, adenocarcinoma; IPF, idiopathic pulmonary fibrosis.
