# Supplementary material for: Extracellular matrix proteins produced by stromal cells in idiopathic pulmonary fibrosis and lung adenocarcinoma
Source: PLoS One. 2021 Apr 27;16(4):e0250109. doi: 10.1371/journal.pone.0250109 (PMC8078755; doi:10.1371/journal.pone.0250109)
Supplement: S2 Fig — (A, B, and C) Spindle shaped cells (black arrowheads) within widened alveolar tips of normal control lung, fibroblast foci of idiopathic pulmonary fibrosis (IPF) lung and stroma of lung adenocarcinoma (ADC) are positive for alpha smooth muscle actin (α-SMA). (D) Some alveolar epithelial cells (black arrows) in normal lung are positive for thyroid transcription factor 1 (TTF-1), which is a marker for type two pneumocytes. (E) Epithelial cells lining fibroblast focus of IPF lung are positive for TTF-1. (F) In lung ADC, cancer cells and cancer stroma are negative for TTF-1. (G) Figure shows cluster of differentiation (CD) 68 positive alveolar macrophages and monocyte lineage cells in normal lung. (H and I) Some cells within the fibroblast focus and cancer stroma are positive for CD68 (white arrowhead), which stains macrophages and monocyte lineage cells. (J, K and L) Figure shows CD31 positive endothelial cells (white arrows) lining capillaries within normal lung, fibroblast foci of IPF lung and stroma of ADC. (M, N and O) Negative controls where primary antibody was substituted with rabbit isotype control, mouse isotype control or phosphate buffered saline (PBS), respectively. Scale bar 50 μm. (PDF) [file pone.0250109.s002.pdf]

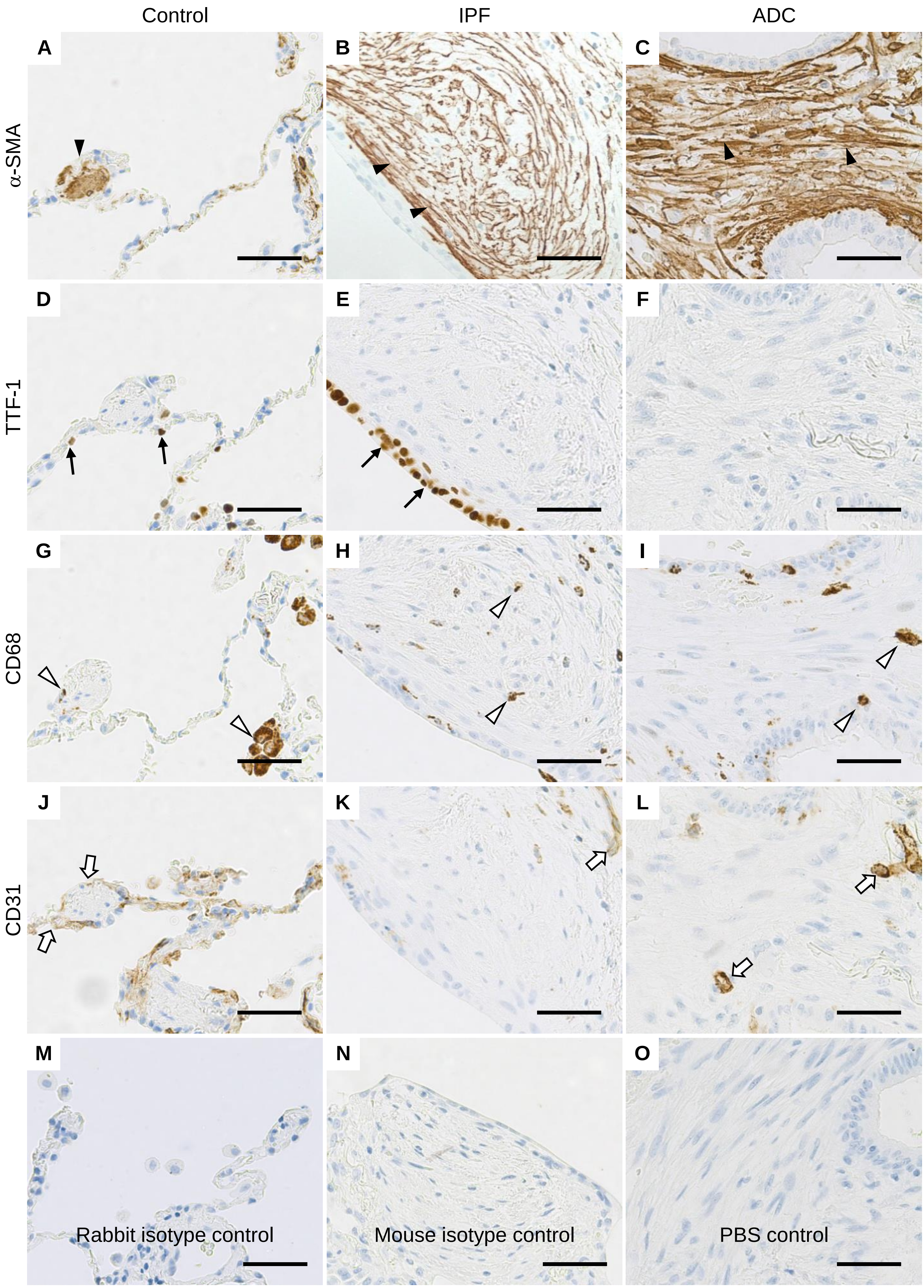

Control
IPF
ADC
α-SMA
TTF-1
CD68
CD31
A
B
C
D
E
F
G
H
I
J
K
L
M
N
O
Rabbit isotype control
Mouse isotype control
PBS control

S2 Fig. Immunohistochemical localization of α-SMA, TTF-1, CD68 and CD31 in normal control lung, IPF and ADC. (A, B, and C) Spindle shaped cells (black arrowheads) within widened alveolar tips of normal control lung, fibroblast foci of idiopathic pulmonary fibrosis (IPF) lung and stroma of lung adenocarcinoma (ADC) are positive for alpha smooth muscle actin (α-SMA). (D) Some alveolar epithelial cells (black arrows) in normal lung are positive for thyroid transcription factor 1 (TTF-1), which is a marker for type two pneumocytes. (E) Epithelial cells lining fibroblast focus of IPF lung are positive for TTF-1. (F) In lung ADC, cancer cells and cancer stroma are negative for TTF-1. (G) Figure shows cluster of differentiation (CD) 68 positive alveolar macrophages and monocyte lineage cells in normal lung. (H and I) Some cells within the fibroblast focus and cancer stroma are positive for CD68 (white arrowhead), which stains macrophages and monocyte lineage cells. (J, K and L) Figure shows CD31 positive endothelial cells (white arrows) lining capillaries within normal lung, fibroblast foci of IPF lung and stroma of ADC. (M, N and O) Negative controls where primary antibody was substituted with rabbit isotype control, mouse isotype control or phosphate buffered saline (PBS), respectively. Scale bar 50 µm.
