# Supplementary material for: Extracellular matrix proteins produced by stromal cells in idiopathic pulmonary fibrosis and lung adenocarcinoma
Source: PLoS One. 2021 Apr 27;16(4):e0250109. doi: 10.1371/journal.pone.0250109 (PMC8078755; doi:10.1371/journal.pone.0250109)
Supplement: S1 Table — (DOCX) [file pone.0250109.s003.docx]

**S1 Table.** **Microarray studies using cultured stromal cells derived from IPF or ADC.**

| **Study** | **Patient samples** | **Control samples** | **Smoking status** | **Microarray platform** |
| --- | --- | --- | --- | --- |
| Vuga, L. J. *et al*. Am J Respir Cell Mol Biol 2009; 41: 583-589. | 4 explants with UIP histology | 3 normal histology lungs from organ donors | Not available | CodeLink UniSet Human I Bioarray |
| Hsu, E. *et al*. Arthritis Rheum. 2011;63: 783-794. | 9 SSc-PF, 9 SSc-PAH, 10 IPF and 6 IPAH from explants | 9 controls from organ donors whose lungs were not used for lung transplantation | 3 SSc-PF, 4 SSc-PAH, 3 IPF, 4 IPAH and 3 controls smokers, 6 SSc-PF, 5 SSc-PAH, 7 IPF, 4 IPAH and 3 controls non-smokers | Illumina HumanRef-8 v3.0 |
| Lindahl, G. E. *et al*. Respir Res 2013; 14: 80. | 3 IPF and 8 SSc-ILD surgical lung biopsies | 10 controls from tumor-free lung resections of patients with lung cancer | 4 SSc-ILDs and 2 IPFs ex-smokers, not available for all control cases | Affymetrix human U133Av2 |
| Peng, R. *et al*. PLoS One 2013; 8: e59348. | 4 rapidly progressing and 6 stable IPF patients | 4 controls from tumor-free lung resections of patients with lung cancer | Controls non-smokers | Affymetrix Human Genome U133 Plus 2.0 |
| Lee, J. U. *et al*. Respir Res 2017; 18: 3-6. | 8 surgical lung biopsies from IPF patients | 4 tumor-free lung resections of patients with lung cancer | Controls and 4 IPFs non-smokers, 4 IPFs ex-smokers | Illumina HumanHT-12 V4.0 |
| Rodriguez, L. R. *et al*. Sci Rep 2018; 8: 3983. | 8 explants from from patients with advanced IPF | 4 controls from donors not suitable for lung transplantation | 6 IPFs ex-smokers, controls non-smokers | Duke Operon Human 36K oligonucleotide array V4.0 |
| Navab, R. *et al*. Proc Natl Acad Sci U S A 2011; 108: 7160-7165. | 15 resected non-small cell lung cancer tumors | 15 tumor-free lung resections of patients with lung cancer | Not available | Affymetrix Human Exon 1.0 ST Array |

ADC, lung adenocarcinoma; IPAH, idiopathic pulmonary arterial hypertension; IPF, idiopathic pulmonary fibrosis; UIP, usual interstitial pneumonia; SSc-ILD, scleroderma associated interstitial lung disease; SSc-PF, scleroderma related pulmonary fibrosis; SSc-PAH, scleroderma related pulmonary arterial hypertension
