# Supplementary material for: Extracellular matrix proteins produced by stromal cells in idiopathic pulmonary fibrosis and lung adenocarcinoma
Source: PLoS One. 2021 Apr 27;16(4):e0250109. doi: 10.1371/journal.pone.0250109 (PMC8078755; doi:10.1371/journal.pone.0250109)
Supplement: S2 Table — (DOCX) [file pone.0250109.s004.docx]

**S2 Table.** **Sequences, annealing temperatures and amplicon sizes of quantitative RT-PCR primers used in this study.**

| **Primer** | | **Sequence 5'-3'** | **T_a_ (°C)** | **Amplicon size (bp)** |
| --- | --- | --- | --- | --- |
| COL4A1 | Forward | CCCCAAAGGTGTTGACGGCT | 61.4 | 126 |
|  | Reverse | AGACCAACTCCAGGCTCTCC |  |  |
| GAPDH | Forward | GAGTCAACGGATTTGGTCGT | 63 | 185 |
|  | Reverse | GACAAGCTTCCCGTTCTCAG |  |  |
| HMBS | Forward | TGCAACGGCGGAAGAAAA | 59 | 66 |
|  | Reverse | AGCTGGCTCTTGCGGGTAC |  |  |
| MMP1 | Forward | TCACAGCTTCCCAGCGACTC | 60 | 128 |
|  | Reverse | CTGGGCCACTATTTCTCCGCT |  |  |
| MMP3 | Forward | TGAGGACACCAGCATGAACC | 63 | 99 |
|  | Reverse | GGACCACTGTCCTTTCTCCTAAC |  |  |
| POSTN | Forward | GGAGACAAAGTGGCTTCCGA | 62.2 | 104 |
|  | Reverse | CCTTCCAGCGTCTCAAAGACT |  |  |
