# Supplementary material for: Extracellular matrix proteins produced by stromal cells in idiopathic pulmonary fibrosis and lung adenocarcinoma
Source: PLoS One. 2021 Apr 27;16(4):e0250109. doi: 10.1371/journal.pone.0250109 (PMC8078755; doi:10.1371/journal.pone.0250109)
Supplement: S3 Table — (DOCX) [file pone.0250109.s005.docx]

**S3 Table.** **Antibodies used for immunohistochemistry in this study.**

| **Antibody** | **Manufacturer** | **Catalogue number** | **Antigen retrieval** | **Dilution** | **Incubation** |
| --- | --- | --- | --- | --- | --- |
| Polyclonal Rabbit anti-Periostin antibody | Abcam, Cambridge, UK | ab14041 | MW in citrate buffer (pH 6) for 15 min | 1:3000 | o/n +4°C |
| Polyclonal Rabbit anti-Collagen IV alpha 1 antibody | Novus Biologicals, Abingdon, UK | NB120-6586 | Pepsin treatment for 30 min at 37°C | 1:75 | 1h RT |
| Monoclonal mouse anti-MMP-1 antibody (clone Ab-1) | Oncogene Research Products, Cambridge, Mass., USA | IM35L | MW in citrate buffer (pH 6) for 15 min | 1:75 | 1h RT |
| Polyclonal rabbit Anti-MMP-3 antibody | Abcam | ab137659 | MW in citrate buffer (pH 6) for 15 min | 1:100 | 1h RT |
| Monoclonal Mouse Smooth Muscle Actin (clone 1A4) | Dako, Glostrup, Denmark | M0851 | MW in Tris-EDTA buffer (pH 9) for 15 min | 1:1000 | 30 min RT |
| Monoclonal Mouse anti-human CD68 (clone PG-M1) | Dako | M0876 | MW in Tris-EDTA buffer (pH 9) for 15 min | 1:300 | 30 min RT |
| Monoclonal Mouse Anti-Thyroid Transcription Factor (clone 8G7G3/1) | Dako | M3575 | MW in Tris-EDTA buffer (pH 9) for 15 min | 1:200 | 30 min RT |
| Monoclonal Mouse Anti-Human CD31 (clone JC70A) | Dako | M0823 | MW in Tris-EDTA buffer (pH 9) for 15 min | 1:100 | 30 min RT |

MW, microwave heat treatment; o/n, overnight; RT, room temperature.
