# Supplementary material for: Extracellular matrix proteins produced by stromal cells in idiopathic pulmonary fibrosis and lung adenocarcinoma
Source: PLoS One. 2021 Apr 27;16(4):e0250109. doi: 10.1371/journal.pone.0250109 (PMC8078755; doi:10.1371/journal.pone.0250109)
Supplement: S4 Table — All up- or down-regulated genes (log2FC higher than 1 or lower than -1) in stromal cells derived from patients with IPF compared to normal control lung. (DOCX) [file pone.0250109.s006.docx]

**S4 Table.** **Differentially expressed genes in IPF compared to control.**

| **Affymetrix probe** | **Gene symbol** | **Description** | **Log_2_FC (IPF vs. Control)** |
| --- | --- | --- | --- |
| 6192_at | *RPS4Y1* | ribosomal protein S4, Y-linked 1 | 4.4525 |
| 9086_at | *EIF1AY* | eukaryotic translation initiation factor 1A, Y-linked | 3.2 |
| 4314_at | *MMP3* | matrix metallopeptidase 3 | 3.095 |
| 3223_at | *HOXC6* | homeobox C6 | 2.765 |
| 3598_at | *IL13RA2* | interleukin 13 receptor, alpha 2 | 2.6525 |
| 11009_at | *IL24* | interleukin 24 | 2.61 |
| 8653_at | *DDX3Y* | DEAD (Asp-Glu-Ala-Asp) box helicase 3, Y-linked | 2.5925 |
| 6275_at | *S100A4* | S100 calcium binding protein A4 | 2.56 |
| 8673_at | *VAMP8* | vesicle-associated membrane protein 8 | 2.38 |
| 8284_at | *KDM5D* | lysine (K)-specific demethylase 5D | 2.325 |
| 8287_at | *USP9Y* | ubiquitin specific peptidase 9, Y-linked | 2.2725 |
| 64595_at | *TTTY15* | testis-specific transcript, Y-linked 15 (non-protein coding) | 2.255 |
| 220_at | *ALDH1A3* | aldehyde dehydrogenase 1 family, member A3 | 2.205 |
| 10457_at | *GPNMB* | glycoprotein (transmembrane) nmb | 2.09 |
| 55531_at | *ELMOD1* | ELMO/CED-12 domain containing 1 | 2.0475 |
| 5420_at | *PODXL* | podocalyxin-like | 2.0225 |
| 4319_at | *MMP10* | matrix metallopeptidase 10 | 2 |
| 375295_at | *LINC01116* | long intergenic non-protein coding RNA 1116 | 1.945 |
| 1116_at | *CHI3L1* | chitinase 3-like 1 (cartilage glycoprotein-39) | 1.92 |
| 5176_at | *SERPINF1* | serpin peptidase inhibitor, clade F (alpha-2 antiplasmin, pigment epithelium derived factor), member 1 | 1.8925 |
| 9120_at | *SLC16A6* | solute carrier family 16, member 6 | 1.855 |
| 4223_at | *MEOX2* | mesenchyme homeobox 2 | 1.85 |
| 100131187_at | *TSTD1* | thiosulfate sulfurtransferase (rhodanese)-like domain containing 1 | 1.8475 |
| 27075_at | *TSPAN13* | tetraspanin 13 | 1.8225 |
| 246126_at | *TXLNGY* | taxilin gamma pseudogene, Y-linked | 1.7325 |
| 9244_at | *CRLF1* | cytokine receptor-like factor 1 | 1.725 |
| 10234_at | *LRRC17* | leucine rich repeat containing 17 | 1.725 |
| 286333_at | *FAM225A* | family with sequence similarity 225, member A (non-protein coding) | 1.645 |
| 2201_at | *FBN2* | fibrillin 2 | 1.6175 |
| 597_at | *BCL2A1* | BCL2-related protein A1 | 1.5975 |
| 23136_at | *EPB41L3* | erythrocyte membrane protein band 4.1-like 3 | 1.59 |
| 6781_at | *STC1* | stanniocalcin 1 | 1.58 |
| 1475_at | *CSTA* | cystatin A (stefin A) | 1.5625 |
| 26050_at | *SLITRK5* | SLIT and NTRK-like family, member 5 | 1.545 |
| 2069_at | *EREG* | epiregulin | 1.53 |
| 4312_at | *MMP1* | matrix metallopeptidase 1 | 1.5 |
| 728215_at | *FAM155A* | family with sequence similarity 155, member A | 1.495 |
| 8989_at | *TRPA1* | transient receptor potential cation channel, subfamily A, member 1 | 1.4675 |
| 948_at | *CD36* | CD36 molecule (thrombospondin receptor) | 1.465 |
| 7980_at | *TFPI2* | tissue factor pathway inhibitor 2 | 1.41 |
| 25893_at | *TRIM58* | tripartite motif containing 58 | 1.3525 |
| 29953_at | *TRHDE* | thyrotropin-releasing hormone degrading enzyme | 1.345 |
| 81849_at | *ST6GALNAC5* | ST6 (alpha-N-acetyl-neuraminyl-2,3-beta-galactosyl-1,3)-N-acetylgalactosaminide alpha-2,6-sialyltransferase 5 | 1.3275 |
| 79191_at | *IRX3* | iroquois homeobox 3 | 1.255 |
| 338376_at | *IFNE* | interferon, epsilon | 1.205 |
| 1300_at | *COL10A1* | collagen, type X, alpha 1 | 1.195 |
| 100131897_at | *FAM196B* | family with sequence similarity 196, member B | 1.1625 |
| 3775_at | *KCNK1* | potassium channel, two pore domain subfamily K, member 1 | 1.1625 |
| 7130_at | *TNFAIP6* | tumor necrosis factor, alpha-induced protein 6 | 1.15 |
| 92737_at | *DNER* | delta/notch-like EGF repeat containing | 1.14 |
| 730755_at | *KRTAP2-3* | keratin associated protein 2-3 | 1.12 |
| 84803_at | *GPAT3* | glycerol-3-phosphate acyltransferase 3 | 1.1175 |
| 9079_at | *LDB2* | LIM domain binding 2 | 1.105 |
| 25903_at | *OLFML2B* | olfactomedin-like 2B | 1.1025 |
| 2921_at | *CXCL3* | chemokine (C-X-C motif) ligand 3 | 1.1 |
| 161198_at | *CLEC14A* | C-type lectin domain family 14, member A | 1.0875 |
| 400043_at | *LOC400043* | uncharacterized LOC400043 | 1.085 |
| 4311_at | *MME* | membrane metallo-endopeptidase | 1.0725 |
| 79746_at | *ECHDC3* | enoyl CoA hydratase domain containing 3 | 1.0675 |
| 646_at | *BNC1* | basonuclin 1 | 1.06 |
| 3592_at | *IL12A* | interleukin 12A | 1.0525 |
| 10631_at | *POSTN* | periostin, osteoblast specific factor | 1.0275 |
| 7078_at | *TIMP3* | TIMP metallopeptidase inhibitor 3 | 1.02 |
| 554202_at | *MIR31HG* | MIR31 host gene | 1.01 |
| 22829_at | *NLGN4Y* | neuroligin 4, Y-linked | 1.01 |
| 7056_at | *THBD* | thrombomodulin | 1.01 |
| 5646_at | *PRSS3* | protease, serine, 3 | 1.0025 |
| 30011_at | *SH3KBP1* | SH3-domain kinase binding protein 1 | 1.0025 |
| 70_at | *ACTC1* | actin, alpha, cardiac muscle 1 | -1.0025 |
| 6664_at | *SOX11* | SRY (sex determining region Y)-box 11 | -1.0025 |
| 1373_at | *CPS1* | carbamoyl-phosphate synthase 1, mitochondrial | -1.005 |
| 10875_at | *FGL2* | fibrinogen-like 2 | -1.0125 |
| 687_at | *KLF9* | Kruppel-like factor 9 | -1.0225 |
| 1282_at | *COL4A1* | collagen, type IV, alpha 1 | -1.025 |
| 11096_at | *ADAMTS5* | ADAM metallopeptidase with thrombospondin type 1 motif, 5 | -1.0325 |
| 29995_at | *LMCD1* | LIM and cysteine-rich domains 1 | -1.0325 |
| 8654_at | *PDE5A* | phosphodiesterase 5A, cGMP-specific | -1.035 |
| 3569_at | *IL6* | interleukin 6 | -1.0375 |
| 5137_at | *PDE1C* | phosphodiesterase 1C, calmodulin-dependent 70kDa | -1.04 |
| 651746_at | *ANKRD33B* | ankyrin repeat domain 33B | -1.0425 |
| 4147_at | *MATN2* | matrilin 2 | -1.045 |
| 633_at | *BGN* | biglycan | -1.0475 |
| 2634_at | *GBP2* | guanylate binding protein 2, interferon-inducible | -1.05 |
| 135293_at | *PM20D2* | peptidase M20 domain containing 2 | -1.0575 |
| 728392_at | *LOC728392* | uncharacterized LOC728392 | -1.06 |
| 57134_at | *MAN1C1* | mannosidase, alpha, class 1C, member 1 | -1.0675 |
| 4908_at | *NTF3* | neurotrophin 3 | -1.0825 |
| 4744_at | *NEFH* | neurofilament, heavy polypeptide | -1.095 |
| 176_at | *ACAN* | aggrecan | -1.0975 |
| 80099_at | *C7orf69* | chromosome 7 open reading frame 69 | -1.0975 |
| 4688_at | *NCF2* | neutrophil cytosolic factor 2 | -1.0975 |
| 4919_at | *ROR1* | receptor tyrosine kinase-like orphan receptor 1 | -1.0975 |
| 5764_at | *PTN* | pleiotrophin | -1.11 |
| 1306_at | *COL15A1* | collagen, type XV, alpha 1 | -1.1125 |
| 5357_at | *PLS1* | plastin 1 | -1.1125 |
| 102724927_at | *LOC102724927* | uncharacterized LOC102724927 | -1.115 |
| 11098_at | *PRSS23* | protease, serine, 23 | -1.1225 |
| 58494_at | *JAM2* | junctional adhesion molecule 2 | -1.125 |
| 29968_at | *PSAT1* | phosphoserine aminotransferase 1 | -1.125 |
| 6387_at | *CXCL12* | chemokine (C-X-C motif) ligand 12 | -1.1475 |
| 1012_at | *CDH13* | cadherin 13 | -1.175 |
| 3815_at | *KIT* | v-kit Hardy-Zuckerman 4 feline sarcoma viral oncogene homolog | -1.18 |
| 4060_at | *LUM* | lumican | -1.18 |
| 771_at | *CA12* | carbonic anhydrase XII | -1.1825 |
| 64399_at | *HHIP* | hedgehog interacting protein | -1.1825 |
| 115701_at | *ALPK2* | alpha-kinase 2 | -1.1925 |
| 3679_at | *ITGA7* | integrin, alpha 7 | -1.1975 |
| 53346_at | *TM6SF1* | transmembrane 6 superfamily member 1 | -1.215 |
| 85480_at | *TSLP* | thymic stromal lymphopoietin | -1.215 |
| 8825_at | *LIN7A* | lin-7 homolog A (C. elegans) | -1.2175 |
| 152742_at | *LINC01085* | long intergenic non-protein coding RNA 1085 | -1.225 |
| 220965_at | *FAM13C* | family with sequence similarity 13, member C | -1.2275 |
| 9249_at | *DHRS3* | dehydrogenase/reductase (SDR family) member 3 | -1.235 |
| 117248_at | *GALNT15* | polypeptide N-acetylgalactosaminyltransferase 15 | -1.235 |
| 26577_at | *PCOLCE2* | procollagen C-endopeptidase enhancer 2 | -1.235 |
| 2202_at | *EFEMP1* | EGF containing fibulin-like extracellular matrix protein 1 | -1.24 |
| 93649_at | *MYOCD* | myocardin | -1.2725 |
| 121512_at | *FGD4* | FYVE, RhoGEF and PH domain containing 4 | -1.275 |
| 283298_at | *OLFML1* | olfactomedin-like 1 | -1.3075 |
| 1794_at | *DOCK2* | dedicator of cytokinesis 2 | -1.3125 |
| 1191_at | *CLU* | clusterin | -1.33 |
| 64798_at | *DEPTOR* | DEP domain containing MTOR-interacting protein | -1.3375 |
| 83716_at | *CRISPLD2* | cysteine-rich secretory protein LCCL domain containing 2 | -1.365 |
| 51200_at | *CPA4* | carboxypeptidase A4 | -1.385 |
| 590_at | *BCHE* | butyrylcholinesterase | -1.4 |
| 1129_at | *CHRM2* | cholinergic receptor, muscarinic 2 | -1.415 |
| 1634_at | *DCN* | decorin | -1.415 |
| 54541_at | *DDIT4* | DNA-damage-inducible transcript 4 | -1.44 |
| 54873_at | *PALMD* | palmdelphin | -1.445 |
| 4162_at | *MCAM* | melanoma cell adhesion molecule | -1.4525 |
| 3045_at | *HBD* | hemoglobin, delta | -1.485 |
| 30061_at | *SLC40A1* | solute carrier family 40 (iron-regulated transporter), member 1 | -1.505 |
| 4133_at | *MAP2* | microtubule-associated protein 2 | -1.515 |
| 6328_at | *SCN3A* | sodium channel, voltage gated, type III alpha subunit | -1.5325 |
| 25801_at | *GCA* | grancalcin, EF-hand calcium binding protein | -1.5525 |
| 440_at | *ASNS* | asparagine synthetase (glutamine-hydrolyzing) | -1.5725 |
| 167681_at | *PRSS35* | protease, serine, 35 | -1.61 |
| 158471_at | *PRUNE2* | prune homolog 2 (Drosophila) | -1.6175 |
| 100505633_at | *LINC01133* | long intergenic non-protein coding RNA 1133 | -1.6375 |
| 84675_at | *TRIM55* | tripartite motif containing 55 | -1.6375 |
| 4256_at | *MGP* | matrix Gla protein | -1.64 |
| 59277_at | *NTN4* | netrin 4 | -1.64 |
| 23767_at | *FLRT3* | fibronectin leucine rich transmembrane protein 3 | -1.65 |
| 339479_at | *BRINP3* | bone morphogenetic protein/retinoic acid inducible neural-specific 3 | -1.6825 |
| 84709_at | *MGARP* | mitochondria-localized glutamic acid-rich protein | -1.76 |
| 7164_at | *TPD52L1* | tumor protein D52-like 1 | -1.7825 |
| 8988_at | *HSPB3* | heat shock 27kDa protein 3 | -1.7975 |
| 57007_at | *ACKR3* | atypical chemokine receptor 3 | -1.86 |
| 4883_at | *NPR3* | natriuretic peptide receptor 3 | -1.88 |
| 23705_at | *CADM1* | cell adhesion molecule 1 | -1.89 |
| 5918_at | *RARRES1* | retinoic acid receptor responder (tazarotene induced) 1 | -1.93 |
| 27063_at | *ANKRD1* | ankyrin repeat domain 1 (cardiac muscle) | -2.1325 |
| 91851_at | *CHRDL1* | chordin-like 1 | -2.22 |
| 4232_at | *MEST* | mesoderm specific transcript | -2.2425 |
| 2167_at | *FABP4* | fatty acid binding protein 4, adipocyte | -2.5225 |
| 55026_at | *TMEM255A* | transmembrane protein 255A | -2.61 |
| 7503_at | *XIST* | X inactive specific transcript (non-protein coding) | -2.64 |

All up- or down-regulated genes (log_2_FC higher than 1 or lower than -1) in stromal cells derived from patients with IPF compared to normal control lung. IPF, idiopathic pulmonary fibrosis; log_2_FC, log_2_ fold change.
