# Supplementary material for: Extracellular matrix proteins produced by stromal cells in idiopathic pulmonary fibrosis and lung adenocarcinoma
Source: PLoS One. 2021 Apr 27;16(4):e0250109. doi: 10.1371/journal.pone.0250109 (PMC8078755; doi:10.1371/journal.pone.0250109)
Supplement: S5 Table — All up- or down-regulated genes (log2FC higher than 1 or lower than -1) in stromal cells derived from patients with IPF compared to ADC. (DOCX) [file pone.0250109.s007.docx]

**S5 Table.** **Differentially expressed genes in IPF compared to ADC.**

| **Affymetrix probe** | **Gene symbol** | **Description** | **Log_2_FC (IPF vs. ADC)** |
| --- | --- | --- | --- |
| 6192_at | *RPS4Y1* | ribosomal protein S4, Y-linked 1 | 4.17 |
| 9086_at | *EIF1AY* | eukaryotic translation initiation factor 1A, Y-linked | 2.9625 |
| 3598_at | *IL13RA2* | interleukin 13 receptor, alpha 2 | 2.62 |
| 8653_at | *DDX3Y* | DEAD (Asp-Glu-Ala-Asp) box helicase 3, Y-linked | 2.4825 |
| 4312_at | *MMP1* | matrix metallopeptidase 1 | 2.435 |
| 4314_at | *MMP3* | matrix metallopeptidase 3 | 2.4025 |
| 8287_at | *USP9Y* | ubiquitin specific peptidase 9, Y-linked | 2.1825 |
| 8284_at | *KDM5D* | lysine (K)-specific demethylase 5D | 2.04 |
| 64595_at | *TTTY15* | testis-specific transcript, Y-linked 15 (non-protein coding) | 1.895 |
| 11009_at | *IL24* | interleukin 24 | 1.8025 |
| 597_at | *BCL2A1* | BCL2-related protein A1 | 1.775 |
| 55531_at | *ELMOD1* | ELMO/CED-12 domain containing 1 | 1.74 |
| 84803_at | *GPAT3* | glycerol-3-phosphate acyltransferase 3 | 1.61 |
| 4319_at | *MMP10* | matrix metallopeptidase 10 | 1.6075 |
| 220_at | *ALDH1A3* | aldehyde dehydrogenase 1 family, member A3 | 1.5975 |
| 6781_at | *STC1* | stanniocalcin 1 | 1.5825 |
| 3223_at | *HOXC6* | homeobox C6 | 1.5775 |
| 100131187_at | *TSTD1* | thiosulfate sulfurtransferase (rhodanese)-like domain containing 1 | 1.545 |
| 246126_at | *TXLNGY* | taxilin gamma pseudogene, Y-linked | 1.515 |
| 7980_at | *TFPI2* | tissue factor pathway inhibitor 2 | 1.475 |
| 5646_at | *PRSS3* | protease, serine, 3 | 1.37 |
| 26050_at | *SLITRK5* | SLIT and NTRK-like family, member 5 | 1.35 |
| 6275_at | *S100A4* | S100 calcium binding protein A4 | 1.345 |
| 9120_at | *SLC16A6* | solute carrier family 16, member 6 | 1.3225 |
| 5672_at | *PSG4* | pregnancy specific beta-1-glycoprotein 4 | 1.3025 |
| 27075_at | *TSPAN13* | tetraspanin 13 | 1.295 |
| 25893_at | *TRIM58* | tripartite motif containing 58 | 1.2875 |
| 8989_at | *TRPA1* | transient receptor potential cation channel, subfamily A, member 1 | 1.22 |
| 29953_at | *TRHDE* | thyrotropin-releasing hormone degrading enzyme | 1.21 |
| 10234_at | *LRRC17* | leucine rich repeat containing 17 | 1.2025 |
| 2921_at | *CXCL3* | chemokine (C-X-C motif) ligand 3 | 1.1975 |
| 338376_at | *IFNE* | interferon, epsilon | 1.195 |
| 10391_at | *CORO2B* | coronin, actin binding protein, 2B | 1.1825 |
| 1901_at | *S1PR1* | sphingosine-1-phosphate receptor 1 | 1.165 |
| 5321_at | *PLA2G4A* | phospholipase A2, group IVA (cytosolic, calcium-dependent) | 1.135 |
| 5420_at | *PODXL* | podocalyxin-like | 1.1175 |
| 2069_at | *EREG* | epiregulin | 1.1025 |
| 7056_at | *THBD* | thrombomodulin | 1.0925 |
| 3776_at | *KCNK2* | potassium channel, two pore domain subfamily K, member 2 | 1.085 |
| 92737_at | *DNER* | delta/notch-like EGF repeat containing | 1.0525 |
| 2201_at | *FBN2* | fibrillin 2 | 1.045 |
| 161198_at | *CLEC14A* | C-type lectin domain family 14, member A | 1.04 |
| 6374_at | *CXCL5* | chemokine (C-X-C motif) ligand 5 | 1.0025 |
| 23767_at | *FLRT3* | fibronectin leucine rich transmembrane protein 3 | -1.005 |
| 1404_at | *HAPLN1* | hyaluronan and proteoglycan link protein 1 | -1.005 |
| 91851_at | *CHRDL1* | chordin-like 1 | -1.01 |
| 54674_at | *LRRN3* | leucine rich repeat neuronal 3 | -1.0125 |
| 25805_at | *BAMBI* | BMP and activin membrane-bound inhibitor | -1.015 |
| 1284_at | *COL4A2* | collagen, type IV, alpha 2 | -1.0175 |
| 2202_at | *EFEMP1* | EGF containing fibulin-like extracellular matrix protein 1 | -1.02 |
| 1794_at | *DOCK2* | dedicator of cytokinesis 2 | -1.0225 |
| 728392_at | *LOC728392* | uncharacterized LOC728392 | -1.0225 |
| 440_at | *ASNS* | asparagine synthetase (glutamine-hydrolyzing) | -1.035 |
| 687_at | *KLF9* | Kruppel-like factor 9 | -1.035 |
| 5521_at | *PPP2R2B* | protein phosphatase 2, regulatory subunit B, beta | -1.04 |
| 101929407_at | *PTPRD-AS1* | PTPRD antisense RNA 1 | -1.0425 |
| 4883_at | *NPR3* | natriuretic peptide receptor 3 | -1.045 |
| 1012_at | *CDH13* | cadherin 13 | -1.05 |
| 171024_at | *SYNPO2* | synaptopodin 2 | -1.0525 |
| 4133_at | *MAP2* | microtubule-associated protein 2 | -1.06 |
| 7058_at | *THBS2* | thrombospondin 2 | -1.0675 |
| 11098_at | *PRSS23* | protease, serine, 23 | -1.0725 |
| 51200_at | *CPA4* | carboxypeptidase A4 | -1.075 |
| 1953_at | *MEGF6* | multiple EGF-like-domains 6 | -1.075 |
| 1634_at | *DCN* | decorin | -1.0825 |
| 60494_at | *CCDC81* | coiled-coil domain containing 81 | -1.0925 |
| 10653_at | *SPINT2* | serine peptidase inhibitor, Kunitz type, 2 | -1.0925 |
| 56133_at | *PCDHB2* | protocadherin beta 2 | -1.115 |
| 81606_at | *LBH* | limb bud and heart development | -1.1375 |
| 8630_at | *HSD17B6* | hydroxysteroid (17-beta) dehydrogenase 6 | -1.14 |
| 25801_at | *GCA* | grancalcin, EF-hand calcium binding protein | -1.1475 |
| 1829_at | *DSG2* | desmoglein 2 | -1.1575 |
| 158471_at | *PRUNE2* | prune homolog 2 (Drosophila) | -1.17 |
| 4908_at | *NTF3* | neurotrophin 3 | -1.1725 |
| 1282_at | *COL4A1* | collagen, type IV, alpha 1 | -1.18 |
| 2517_at | *FUCA1* | fucosidase, alpha-L- 1, tissue | -1.18 |
| 102724927_at | *LOC102724927* | uncharacterized LOC102724927 | -1.19 |
| 3490_at | *IGFBP7* | insulin-like growth factor binding protein 7 | -1.1925 |
| 3569_at | *IL6* | interleukin 6 | -1.2325 |
| 387763_at | *C11orf96* | chromosome 11 open reading frame 96 | -1.245 |
| 10085_at | *EDIL3* | EGF-like repeats and discoidin I-like domains 3 | -1.2475 |
| 10335_at | *MRVI1* | murine retrovirus integration site 1 homolog | -1.25 |
| 22998_at | *LIMCH1* | LIM and calponin homology domains 1 | -1.2575 |
| 10699_at | *CORIN* | corin, serine peptidase | -1.2675 |
| 30061_at | *SLC40A1* | solute carrier family 40 (iron-regulated transporter), member 1 | -1.275 |
| 29995_at | *LMCD1* | LIM and cysteine-rich domains 1 | -1.285 |
| 3045_at | *HBD* | hemoglobin, delta | -1.29 |
| 100507632_at | *LINC00968* | long intergenic non-protein coding RNA 968 | -1.2925 |
| 590_at | *BCHE* | butyrylcholinesterase | -1.2975 |
| 339479_at | *BRINP3* | bone morphogenetic protein/retinoic acid inducible neural-specific 3 | -1.2975 |
| 8076_at | *MFAP5* | microfibrillar associated protein 5 | -1.2975 |
| 4060_at | *LUM* | lumican | -1.31 |
| 115701_at | *ALPK2* | alpha-kinase 2 | -1.315 |
| 5947_at | *RBP1* | retinol binding protein 1, cellular | -1.315 |
| 117248_at | *GALNT15* | polypeptide N-acetylgalactosaminyltransferase 15 | -1.3175 |
| 57007_at | *ACKR3* | atypical chemokine receptor 3 | -1.3325 |
| 1306_at | *COL15A1* | collagen, type XV, alpha 1 | -1.3375 |
| 2304_at | *FOXE1* | forkhead box E1 | -1.3575 |
| 72_at | *ACTG2* | actin, gamma 2, smooth muscle, enteric | -1.36 |
| 3815_at | *KIT* | v-kit Hardy-Zuckerman 4 feline sarcoma viral oncogene homolog | -1.44 |
| 58494_at | *JAM2* | junctional adhesion molecule 2 | -1.465 |
| 23213_at | *SULF1* | sulfatase 1 | -1.49 |
| 94274_at | *PPP1R14A* | protein phosphatase 1, regulatory (inhibitor) subunit 14A | -1.4975 |
| 283298_at | *OLFML1* | olfactomedin-like 1 | -1.5125 |
| 93649_at | *MYOCD* | myocardin | -1.535 |
| 59_at | *ACTA2* | actin, alpha 2, smooth muscle, aorta | -1.5725 |
| 167681_at | *PRSS35* | protease, serine, 35 | -1.5825 |
| 9022_at | *CLIC3* | chloride intracellular channel 3 | -1.5875 |
| 100505633_at | *LINC01133* | long intergenic non-protein coding RNA 1133 | -1.5875 |
| 3357_at | *HTR2B* | 5-hydroxytryptamine (serotonin) receptor 2B, G protein-coupled | -1.5975 |
| 4256_at | *MGP* | matrix Gla protein | -1.6425 |
| 7164_at | *TPD52L1* | tumor protein D52-like 1 | -1.6725 |
| 84709_at | *MGARP* | mitochondria-localized glutamic acid-rich protein | -1.735 |
| 59277_at | *NTN4* | netrin 4 | -1.7425 |
| 8988_at | *HSPB3* | heat shock 27kDa protein 3 | -1.755 |
| 23705_at | *CADM1* | cell adhesion molecule 1 | -1.8175 |
| 2167_at | *FABP4* | fatty acid binding protein 4, adipocyte | -1.8225 |
| 64798_at | *DEPTOR* | DEP domain containing MTOR-interacting protein | -1.9325 |
| 1301_at | *COL11A1* | collagen, type XI, alpha 1 | -1.9725 |
| 70_at | *ACTC1* | actin, alpha, cardiac muscle 1 | -2.0225 |
| 57419_at | *SLC24A3* | solute carrier family 24 (sodium/potassium/calcium exchanger), member 3 | -2.16 |
| 4232_at | *MEST* | mesoderm specific transcript | -2.175 |
| 55026_at | *TMEM255A* | transmembrane protein 255A | -2.26 |
| 7503_at | *XIST* | X inactive specific transcript (non-protein coding) | -2.5475 |
| 27063_at | *ANKRD1* | ankyrin repeat domain 1 (cardiac muscle) | -2.86 |

All up- or down-regulated genes (log_2_FC higher than 1 or lower than -1) in stromal cells derived from patients with IPF compared to ADC. ADC, lung adenocarcinoma; IPF, idiopathic pulmonary fibrosis; log_2_FC, log_2_ fold change
