# Supplementary material for: Extracellular matrix proteins produced by stromal cells in idiopathic pulmonary fibrosis and lung adenocarcinoma
Source: PLoS One. 2021 Apr 27;16(4):e0250109. doi: 10.1371/journal.pone.0250109 (PMC8078755; doi:10.1371/journal.pone.0250109)
Supplement: S6 Table — All up- or down-regulated genes (log2FC higher than 1 or lower than -1) in stromal cells derived from ADC compared to normal control lung. (DOCX) [file pone.0250109.s008.docx]

**S6 Table.** **Differentially expressed genes in ADC compared to control.**

| **Affymetrix probe** | **Gene symbol** | **Description** | **Log_2_FC (ADC vs. Control)** |
| --- | --- | --- | --- |
| 8673_at | *VAMP8* | vesicle-associated membrane protein 8 | 2.275 |
| 1116_at | *CHI3L1* | chitinase 3-like 1 (cartilage glycoprotein-39) | 2.15 |
| 81849_at | *ST6GALNAC5* | ST6 (alpha-N-acetyl-neuraminyl-2,3-beta-galactosyl-1,3)-N-acetylgalactosaminide alpha-2,6-sialyltransferase 5 | 2.12 |
| 1475_at | *CSTA* | cystatin A (stefin A) | 1.97 |
| 948_at | *CD36* | CD36 molecule (thrombospondin receptor) | 1.8475 |
| 10631_at | *POSTN* | periostin, osteoblast specific factor | 1.7 |
| 9244_at | *CRLF1* | cytokine receptor-like factor 1 | 1.4725 |
| 10457_at | *GPNMB* | glycoprotein (transmembrane) nmb | 1.3575 |
| 1545_at | *CYP1B1* | cytochrome P450, family 1, subfamily B, polypeptide 1 | 1.3275 |
| 1301_at | *COL11A1* | collagen, type XI, alpha 1 | 1.325 |
| 57419_at | *SLC24A3* | solute carrier family 24 (sodium/potassium/calcium exchanger), member 3 | 1.315 |
| 8076_at | *MFAP5* | microfibrillar associated protein 5 | 1.3075 |
| 7078_at | *TIMP3* | TIMP metallopeptidase inhibitor 3 | 1.29 |
| 57722_at | *IGDCC4* | immunoglobulin superfamily, DCC subclass, member 4 | 1.2325 |
| 1404_at | *HAPLN1* | hyaluronan and proteoglycan link protein 1 | 1.22 |
| 6275_at | *S100A4* | S100 calcium binding protein A4 | 1.215 |
| 84419_at | *C15orf48* | chromosome 15 open reading frame 48 | 1.1975 |
| 3223_at | *HOXC6* | homeobox C6 | 1.1875 |
| 375295_at | *LINC01116* | long intergenic non-protein coding RNA 1116 | 1.175 |
| 5176_at | *SERPINF1* | serpin peptidase inhibitor, clade F (alpha-2 antiplasmin, pigment epithelium derived factor), member 1 | 1.155 |
| 1829_at | *DSG2* | desmoglein 2 | 1.1475 |
| 3357_at | *HTR2B* | 5-hydroxytryptamine (serotonin) receptor 2B, G protein-coupled | 1.1225 |
| 57482_at | *KIAA1211* | KIAA1211 | 1.1225 |
| 4223_at | *MEOX2* | mesenchyme homeobox 2 | 1.1025 |
| 387763_at | *C11orf96* | chromosome 11 open reading frame 96 | 1.035 |
| 70_at | *ACTC1* | actin, alpha, cardiac muscle 1 | 1.02 |
| 84870_at | *RSPO3* | R-spondin 3 | 1.02 |
| 7292_at | *TNFSF4* | tumor necrosis factor (ligand) superfamily, member 4 | 1.02 |
| 26996_at | *GPR160* | G protein-coupled receptor 160 | -1.0275 |
| 5918_at | *RARRES1* | retinoic acid receptor responder (tazarotene induced) 1 | -1.035 |
| 1129_at | *CHRM2* | cholinergic receptor, muscarinic 2 | -1.0525 |
| 176_at | *ACAN* | aggrecan | -1.055 |
| 26577_at | *PCOLCE2* | procollagen C-endopeptidase enhancer 2 | -1.1325 |
| 84675_at | *TRIM55* | tripartite motif containing 55 | -1.1825 |
| 91851_at | *CHRDL1* | chordin-like 1 | -1.21 |
| 5649_at | *RELN* | reelin | -1.6275 |

All up- or down-regulated genes (log_2_FC higher than 1 or lower than -1) in stromal cells derived from ADC compared to normal control lung. ADC, lung adenocarcinoma; log_2_FC, log_2_ fold change.
